# Living at the edge of an interface area in Zimbabwe: cattle owners, commodity chain and health workers’ awareness, perceptions and practices on zoonoses

**DOI:** 10.1186/s12889-016-2744-3

**Published:** 2016-01-28

**Authors:** B. M. Gadaga, E.M.C. Etter, B. Mukamuri, K. J. Makwangudze, D. M. Pfukenyi, G. Matope

**Affiliations:** 1 Department of Veterinary Technical Services, Diagnostics and Research Branch -Central Veterinary Laboratory, Box CY 551, Harare, Zimbabwe; 2 UR AGIRs, Department Environment and Societies, CIRAD, Montpellier, France; 3 Epidemiology Section Department of Production Animal Studies, Faculty of Veterinary Science, University of Pretoria, Private Bag X04, Onderstepoort, 0110 South Africa; 4 Centre for Applied Social Sciences, Faculty of Social Studies, University of Zimbabwe, P.O. Box MP 167, Harare, Zimbabwe; 5 Department of Veterinary Field Services, Chiredzi District Office Box 191, Chiredzi, Zimbabwe; 6 Department of Clinical Veterinary Studies, Faculty of Veterinary Science, University of Zimbabwe, P. O. Box MP 167, Harare, Zimbabwe; 7 Department of Paraclinical Veterinary Studies, Faculty of Veterinary Science, University of Zimbabwe, P. O. Box MP 167, Harare, Zimbabwe

**Keywords:** Cattle owners, Awareness, Zoonoses, Risk perception, Interface, Wildlife

## Abstract

**Background:**

In the great Limpopo transfrontier conservation area (GLTFCA), there is an increased interface between wildlife and domestic animals, because rural households move their cattle into the game park in search of grazing and watering resources. This creates opportunities for inter-species transmission of infectious diseases, including zoonoses like brucellosis and tuberculosis, which may also pose a health risk to the local rural communities. This study investigated the awareness, perceptions and practices on zoonoses amongst rural cattle owners, commodity chain- and health-workers in three different localities around Gonarezhou National Park (GNP), Zimbabwe, where the interface between wild and domestic animals varies.

**Methods:**

A cross-sectional study was conducted in Malipati, Chikombedzi and Chiredzi that are considered to be high-, medium- and low-domestic animal-wildlife interface areas, respectively. Data was collected from cattle owners, commodity chain and health-workers using a semi-structured questionnaire. To determine the public health risk of food-borne zoonoses, their practices with regard to meat and milk consumptions, and measures they take to prevent exposure to infections were assessed. Data were analyzed using descriptive statistics and principal component analysis.

**Results:**

Most respondents (52.8 %, 102/193) were cattle owners, followed by health (30.1 %, 58/193) and lastly commodity chain workers (17.1 %, 33/193). Overall 67.4 % (130/193) of the respondents were aware of zoonoses with respective 48, 81.8, and 93.1 % of cattle owners, commodity chain, and health workers, being aware. Significantly more cattle owners (*P* < 0.05) from medium and low interface areas were aware of zoonoses compared to those from high interface areas. All categories of respondents cited anthrax (69.2 %), rabies (57.7 %), tuberculosis (41.5 %) and brucellosis (23.9 %) as important zoonoses. About half (46.1 %; 89/193) of the respondents perceive wildlife as important reservoirs of zoonoses. High proportions 98.4 % (190/193) and 96.4 % (186/193) of the respondents indicated that they consume meat and milk, respectively. Access to game meat and milk from informal markets was closely associated with consumption of raw meat and milk.

**Conclusions:**

Fewer cattle owners from a high interface area of Malipati are aware of zoonoses compared to other areas due to combined effects of limited education and other factors disadvantaging these marginalised areas. This may increase their risk of exposure to zoonoses, considering that consumption of raw meat and milk is common. Thus, awareness campaigns may reduce the public health impact of zoonoses at the interface.

## Background

Infections that are naturally transmissible from vertebrate animals to humans and vice versa are classified as zoonoses [1]. This class of diseases has been the principal source of emerging health risks and it is estimated that zoonotic pathogens have accounted for more than 60 % of emerging infectious diseases during the past six decades [2]. In addition to having potentially catastrophic impacts on human health and life, zoonotic diseases are associated with significant economic losses to the affected economies as a whole [2]. Zoonotic diseases have both direct and indirect effects on livestock health and production [3]. Indirect effects include the risk of human disease, the economic impact on livestock producers through barriers to trade, the costs associated with control programmes, the increased cost of marketing produce to ensure they are safe for human consumption and the loss of markets because of decreased consumer confidence [4, 5].

Developing countries such as Zimbabwe still have problems regarding the control of zoonoses, mainly due to lack of adequate infrastructure and resources for disease surveillance. Poverty and lack of awareness of zoonoses lead to many people, especially from rural areas, accessing commodities such as fresh un-pasteurized milk and un-inspected meat from domestic animals on the informal food markets. Reduced control of animal movement, lack of systematic and verifiable animal identification (VAI) and product traceability systems, inadequacy of veterinary services to coordinate control of animal diseases and minimal or non-existent inter-sectoral collaboration between the Department of Veterinary and Livestock Development and the providers of human health services further compound the challenges related to control of zoonoses [6].

Shirima et al. [7] and John et al. [8] documented that the risk of zoonoses would increase or decrease, in the various livestock keeping systems and to the public as a whole depending on their awareness, knowledge, attitude and perceptions of zoonoses. Whilst a few studies have been conducted to assess the local pet owners’ [9], dairy farmers’ [10] and rural cattle owners’ [11] awareness and perceptions of zoonoses, there is lack of information on the awareness and perceptions of zoonoses among rural cattle owners, and commodity chain players, and health workers living around the frontiers of human-domestic animal-wildlife interface areas.

A study of the awareness and perceptions of zoonoses amongst cattle owners, particularly at the interface areas where interspecies sharing of infections, including the risk of transmission from animals to humans is possible [12], may help to mitigate the impact of zoonoses in people living at the edges of transfrontier conservation areas (TFCA). In these human-domestic animal-wildlife interface areas, it is also important for health workers (both veterinary and medical) and the key commodity chain players to be aware of zoonoses, including the risk they pose and how they are transmitted, for them to make informed decisions about their control and prevention of exposure to humans [10, 13].

In 2008, the first isolation of *Mycobacterium bovis* in buffaloes from Gonarezhou National Park (GNP) was reported with a strong assumption of epidemiological link with the Kruger National Park [14]. Brucellosis was also detected in cattle at a seroprevalence of 9.9 % [6] and in some buffalo herds [15]. The importance of the interface among humans, domestic animals and wildlife in this region revealed a high potential for transmission of zoonoses. Thus, this study was initiated to assess awareness and perceptions of zoonoses, and management practices to prevent or minimise exposure to zoonotic infections amongst cattle owners, commodity chain and health workers in these interface areas. The study further explores human behaviour and practices in order to establish the major transmission risks of zoonoses and to target the best applicable means to educate and disseminate information in order to reduce the risk of transmission of zoonotic infections. It is envisaged that this research information would facilitate the development of effective joint policies and guidelines by the veterinary and medical departments and help in the “one health” initiatives for control of zoonoses.

## Methods

### Study areas and population

The study was conducted in Chiredzi district located in the south east lowveld (SEL) of Zimbabwe. Part of Chiredzi district includes the Gonarezhou National Park (GNP) which belongs to the Great Limpopo Transfrontier Conservation Areas (GLTFCA) which was established in 2002, and also includes the Kruger National Park and Limpopo National Park in South Africa and Mozambique, respectively. The SEL falls in agro-ecological region V which is semi –arid with a mean temperature of 21.3 °C and receives an annual rainfall of approximately 541 mm [16] that is often erratic and ephemeral, making the area only suitable for extensive animal farming and unsuitable for crop agriculture.

**Fig. 1:**
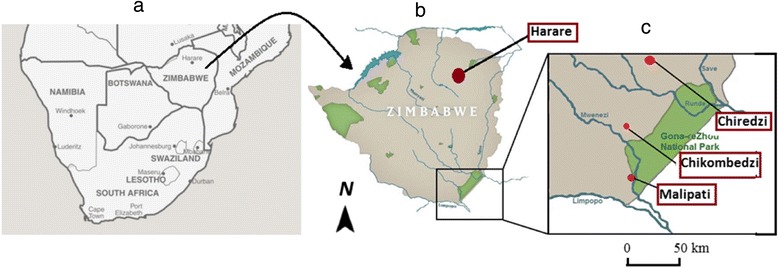
Location of the survey sites in Zimbabwe (**a**) Southern Africa, **b** Zimbabwe, **c** survey areas in the South Eastern Lowveld of Zimbabwe

Chiredzi district is divided into urban/peri-urban and rural areas with a human population of about 30 594 and 276 842, respectively [17]. The cattle population is approximately 189 311, with the majority 169 283 (over 89 %) of them kept in rural areas [18]. Due to geographical, socio-ecological variations in the district, the study areas were selected in relation to their proximity to GNP, and hence on the intensity or probability of interaction of human and domestic animals with wildlife. Thus, the study areas, (Fig. 1) were drawn to represent one of the areas described below.

### High interface area: Malipati

The domestic animal-wildlife interface was defined as a direct physical sharing of the same space at the same time or indirect contact through soil, forage and water with which another animal had recently been in contact and left bodily secretions [15, 19]. Malipati village (22° 04’ S, 31° 25’ E) is located in Sengwe rural areas at the southern periphery of the GNP and in some sections, even included areas of the park. In some of these areas, cattle get access and graze into the park [20] making contact with wildlife highly likely. The veterinary fences that were erected on the boundaries of the national park for control of foot and mouth disease by separating contact between cattle and buffalo, have been damaged extensively in some areas over the past decade. The break-down of the fence has been attributed partly to illegal wildlife hunting activities or domestic animals breaking into the park in search of grazing and water, as the resource gradient between the communal and protected areas increasingly becomes steeper. Due to budgetary constraints following the economic recession that affected the country since 2000, the Department of Veterinary Services was not able to repair some of the broken fences, and hence, this allowed permanent interface between domestic animals and wildlife in certain areas of the park and the adjacent rural areas. The indigenous people that were settled in this area after 1956 are the Ndebele, Shangaan and Karanga (Shona) who have very low income obtained either from agriculture or other activities [21]. Some ethnic differences exist, where, for instance the Shangaan are cattle keepers, who usually have more than 20 animals per family unit as their livelihood depends on cattle, while the Karangas depend on crop agriculture, which often fails due to frequent droughts.

### Medium interface area: Chikombedzi

The rural areas of Chikombedzi (21° 4’S, 31° 20’ E) are located at the south western direction and approximately 15–20 km away from the GNP. These rural areas are separated from the protected areas of the GNP by a fence but in practice this allows possible contact between cattle and wildlife. The rural areas are characterized by small land sizes of approximately 3.7 ha per family unit. Similar to Malipati, crop agriculture is not reliable due to frequent droughts but livestock ownership is common, even though about 20 % of the population keep 10 or more animals [21].

### Low interface area: Chiredzi peri-urban areas

The urban Chiredzi (21° 02’S, 31° 40’E) and peri-urban areas are located approximately 50 km away from GNP. We considered these areas to be low interface because cattle-wildlife contact was believed to be minimal or non-existent. Prior to the year 2000, these areas were designated for commercial cattle ranching, but some of the land was acquired for resettling people on small scale farms (approx. 6 ha each). Most of these cattle owners were from urban Chiredzi and had higher family income compared to those from Malipati. However, they tended to keep smaller herd sizes compared to the other areas with fewer than 20 % of the population keeping 10 or more animals.

### Study design

A cross-sectional study to investigate the awareness and perceptions of zoonoses amongst cattle owners, commodity chain and health workers (both veterinary and medical) was conducted between June 2012 and May 2013. Commodity chain respondents included processors and retailers of meat and milk that were selected from butchery, supermarket and abattoir operators. Since the study was designed to address issues related to awareness perceptions and practices, it was believed (by us) that the inclusion of commodity chain and health workers was vital to study perceptions and practices that may help to mitigate the risk of zoonoses in rural communities.

Questionnaires were developed to assess awareness, perceptions, and practices towards zoonoses. To ease data processing, minimize variation, and improve precision of responses, the questionnaires comprised mainly of closed ended (categorical) questions. The questionnaire was pre-tested on 37 randomly selected individuals that included cattle owners, commodity chain, animal and human health workers and any questions that were noted to be ambiguous were subsequently revised before a large-scale interview. The questionnaires were administered by the principal investigator with the assistance of veterinary and medical officers, using where necessary the three vernacular languages (Ndebele, Shangaan and Shona) that are spoken in the areas, and using a trained interpreter for some of the respondents. To make sure the interviewers were able to elicit effective information from the interviewees in a standardized form, a short training course (three days) was conducted for the interviewers by the principal investigator (B.M. Gadaga) and one of the senior authors (B M Mukamuri) on issues of questionnaire design, setting of questions and their evaluation, and the approaches to administering a questionnaire in a face-to-face interview. The questionnaire was designed to capture general demographic data such as age, gender, occupation, household size, religion and highest level of education attained; commodity (milk and meat) consumption habits; general awareness of zoonoses and awareness of specific zoonotic diseases. Focus of the questionnaire with regards to commodity consumption habits (practices) related to source, frequency, habit (of consuming raw, cooked or boiled meat and milk, respectively) and the reason for the habit. With regards to awareness of zoonoses, key information gathered included awareness of zoonoses, sources of information, listing and ranking of zoonoses, awareness of the mode of transmission, their perceived role of wildlife in transmission of zoonoses, and whether or not they received continuing education on zoonoses. Information was also sought on their views pertaining to the importance of continuing education on zoonoses, which sector should play a leading role in educating them on zoonoses and the reasons for their suggested choice. Specific questions on the awareness of the aetiology, transmission and behaviour predisposing them to exposure to brucellosis and tuberculosis were also included.

### Questionnaire survey

For selection of cattle owners, a two stage selection process was used where first, dip-tanks (for dipping cattle against ticks) were selected randomly, and then cattle owners from each of the selected dip tanks. In Zimbabwe, animal health regulations compel all cattle owners in rural communities to dip their cattle weekly during the rainy season and fortnightly during the dry season for control of ticks and tick-borne diseases [11]. For this reason, the Government, through the Department of Veterinary Services, have constructed communal dip tanks (plunge dip tanks) in all rural animal health centres which are accessible by all cattle owners. Since current records indicate that over 95 % of cattle owners dip their cattle (DVS Annual Reports), we believed the dipping attendance not to be a significant source of selection bias for the cattle owners in any locality. Therefore, due to easy access to cattle owners during the dipping sessions, dip tanks were selected as the sampling frames in the study areas [11]. There were four, seven and four dip tanks that were selected from the low, medium and high interface areas, respectively. At least six farmers per dip tank were selected randomly during the dipping session from a list provided by the local Veterinary Services officer. This gave an estimated sample size of 5 % of stock owners in the study area. To administer the questionnaire, the randomly selected cattle owners or representatives of the owners (interviewees) were interrogated (interviewed) individually for approximately 20–30 min where they were guided through each of the questions and the responses were recorded on the questionnaire by the interviewer. As per cultural norm if the head of household was not at the dip tank but selected for the study, an arrangement was made for a follow up questionnaire interview at their homestead. Study eligibility was based on willingness to be interviewed and being a household head or spouse or a person in-charge of the household aged eighteen years and above in the absence of the household head and the spouse in the case of cattle owners.

With regards to commodity chain workers, the major actors in the meat and milk supply chain were targeted. All abattoirs operating in the Chiredzi district and the two major retail chains/supermarkets serving urban and peri-urban areas were also recruited for the study given the volumes of meat and milk they sell. All butcheries in the medium and high interface areas were also targeted for sampling and there were only two butcheries in the medium interface and none in the high interface. To improve quality of data collected, owners or management were interviewed as they were accountable for all activities at their respective outlets. They also made management decisions as to where and how their commodities are distributed in the communities, some information which may not be obtained from a random sample of abattoir or retail shop employees. In the case of medical health workers, especially in the low and medium interface areas, workers were selected randomly from those available. In the high interface areas, due to low numbers, all workers that were available when the rural health centre was visited were interviewed. Preference was given to health centres serving the cattle owners who were interviewed. A total of 32 medical health workers were interviewed. All the study areas had a total of twenty six (26) veterinary health workers in post and because they could be easily accessed all these were interviewed, giving a total of 58 health workers recruited for the study.

### Statistical analysis

Data recording and edits were done using Microsoft Excel®. Statistical analysis was carried out using Stata SE version 11 for windows (Stata Corp., Texas, USA) to generate descriptive statistics (frequencies/proportions) related to awareness on zoonoses. The data on awareness, perceptions and practices were analyzed with respect to the different groups of the respondents and their areas of origin (0 = high interface; 1 = medium interface; 2 = low interface). For cattle owners, their level of education received (0 = received no high school certificate of education; 1 = received high school certificate of education) was perused according to the areas and the type of religion (0 = traditional; 1 = Christian; 2 = non-believers). Their perceptions (their understanding) on the how the different zooneses were transmitted and the risk factors for their transmission were tabulated. Fisher’s exact Chi-square (*χ* ^2^) test was used to measure associations between categories and values of *P* < 0.05 were considered as significant.

Further evaluation of awareness to zoonoses was performed using the principal component analysis of the FactoMineR package [22], a package for multivariate data analysis with R [23]. The variables were projected on planes constructed with axes. Each axis was a linear combination of the variables that accounted for part of the variability of the whole set of data. The relative importance of each component was expressed by variance (eigenvalue) of its projection or by the proportion of the variance expressed. Close proximity or superimposition in the same quadrant of the variable factors (related to awareness, perceptions or practices) and the groups of individual respondents suggested that the individuals and the variables were positively correlated while position on opposite quadrants of the plane suggested a negative correlation. Interpretation of the individuals factor map was also made by comparing the position of the individuals in the different quadrants with the position of the variables in the same or opposite quadrants on the variables factor map.

## Results

### General characteristics of respondents

A total of 193 respondents; 102 cattle owners (52.8 %), 33 commodity chain (17.1 %), and 58 (30.1 %) health workers were interviewed and 77.2 % (149/193) were males, while 22.8 % (44/193) were females, distributed as; 10.4, 9.3 and 3.1 % cattle owners, health and commodity chain workers, respectively. Most respondents (89.1 %; 172/193) were aged 30 years and above and 69.9 % (135/193) had attained secondary education (high school certificate) and beyond. Of these, 44.1 % (45/102), 97 % (32/33) and 100 % of the cattle owners, commodity chain and health workers, respectively had received at least secondary education. For the cattle owners, 32.3 % (10/31), 45 % (18/40) and 54.8 % (17/31) of the respondents from Malipati (high interface), Chikombedzi (medium) and Chiredzi (low), respectively had received secondary education but the difference was not significant (*P* = 0.21). Their level of education was not influenced by the type of religion (*P* = 0.072), where 22.2, 46.4 and 40 % of traditional believers, Christians and non-believers, respectively had received secondary education.

### General awareness of zoonoses

Table 1 shows the location of the three categories of the respondents and the proportion that was aware of zoonoses. Overall, when asked generally on their awareness of zoonoses, 67.4 % (130/193) of the respondents were aware. Significantly more cattle owners (*P* < 0.05) from medium and low interface areas were aware of zoonoses compared to those from high interface areas (Table 1). A significantly higher percentage of health (93.1 %, *P* < 0.001) and commodity chain workers (81.8 %, *P* = 0.01) were aware of zoonoses compared to cattle owners (48.0 %) (Table 1).

**Table 1:** The number and proportions of zoonoses awareness of farmers, commodity chain-, and health workers interviewed

	Respondent category								
	Cattle owners			Commodity chain workers			Health workers		
Interface category/Area	Total interviewed	Number aware	% aware (95 % CI)	Total interviewed	Number aware	% aware (95 % CI)	Total interviewed	Number aware	% aware (95 % CI)
High/Malipati	31	8	25.8^a^ (12.5–44.9)	0	-	-	12	11	91.7^a^ (59.8–99.6)
Medium/Chikombedzi	40	21	52.5^b^ (36.3–68.2)	2	2	100	11	10	90.9^a^ (57.1–99.5)
Low/Chiredzi	31	20	64.5^b^ (45.4–80.2)	31	25	80.6 (61.9–91.9)	35	33	94.3^a^ (79.5–99.0)
Overall	102	49	48.0 (38.1–58.1)	33	27	81.8 (63.9–92.4)	58	54	93.1 (82.5–97.8)

Proportions with different superscripts (^a, b^) in the same colum are significantly different at P<0.05

Table 2 lists the most cited zoonoses by the respondents. Overall, of the respondents who were aware of zoonoses, most named anthrax (69.2 %), rabies (57.7 %), bovine tuberculosis (bTB) (41.5 %) and brucellosis (23.9 %). Except for anthrax, awareness of named zoonoses differed significantly between respondent categories. Significantly (*P* < 0.01) more health workers named rabies and bTB compared to commodity chain workers and cattle owners. Brucellosis was named by a significantly (*P* < 0.05) higher percentage of both cattle owners and health workers compared to commodity chain workers. Of the respondents who named bTB, only 34.7 and 34.2 % of them were aware of its aetiology and zoonotic importance, respectively, while less than a fifth (16.1 %) were aware of its preventive and control measures. With regard to brucellosis, 34.7 and 32.1 % were aware of its aetiology and zoonotic implications, respectively. A significantly (*P < 0.05*) higher proportion (27.5 %) of respondents was aware of the preventive and control measures for brucellosis compared to bTB.

**Table 2:** Summary of zoonoses named according to respondent category

	Disease named							
	Anthrax		Rabies		Bovine tuberculosis		Brucellosis	
Respondent category	Number naming	% naming (95 % CI)	Number naming	% naming (95 % CI)	Number naming	% naming (95 % CI)	Number naming	% naming (95 % CI)
Cattle owners (*n* = 49)	31	63.3^a^ (48.3–76.2)	22	44.9^a^ (30.9–59.7)	17	34.7^a^ (22.1–49.7)	13	26.5^a^ (15.4–41.3)
Commodity chain workers (*n* = 27)	19	70.4^a^ (49.7–85.5)	13	48.2^a^ (29.2–67.7)	5	18.5^a^ (7.0–38.8)	1	3.7^b^ (0.2–20.9)
Health workers (*n* = 54)	40	74.1^a^ (60.1–84.6)	40	74.1^b^ (60.1–84.6)	32	59.3^a^ (45.1–72.1)	17	31.5^a^ (19.9–45.7)
Overall (*n* = 130)	90	69.2 (60.4–76.9)	75	57.7 (48.7–66.2)	54	41.5 (33.1–50.5)	31	23.9 (17.0–32.3)

Proportions in the same column with different superscripts (^a, b^) are significantly different at P<0.05

Low proportions of respondents that were able to name other relevant zoonoses were as follows; avian and swine influenza (10.0 %), porcine and bovine cysticercosis (tapeworms) (3.1 %) and trypanosomosis (1.5 %). Foot and mouth disease, blackleg and vector-borne diseases such as malaria were wrongly cited as zoonoses by some respondents, including health workers.

About half of the respondents (46.1 %) perceived wildlife to be reservoirs of zoonoses. A low proportion (21.2 %) of the respondents received education on zoonoses with 85.4 % of them citing veterinarians as the source of information. However, a few respondents (13.5 %) cited non-Governmental Organisations and community health workers as providers of education on zoonoses. Of the health workers, only 36.2 % received continuing education on zoonoses.

## Meat and milk eating habits

Table 3 shows the respondents’ perceptions on the different methods of transmission of zoonoses, their consumption habits (practices) for milk and meat and their responses on possible measures to prevent zoonoses. Overall, 36.8, 23.3 and 63.7 % of the respondents indicated that zoonotic infections are acquired through contact, biting by infected animals and consumption of contaminated animal products, respectively. High proportions; 98.4 % (190/193) and 96.4 % (186/193) of the respondents indicated that they consume meat and milk, respectively, with 16.6 and 41.5 % of them consuming raw meat and raw milk (Table 3). Generally, consumption of game meat was noted to be high (66.8 %) among the respondents with cattle owners recording the highest percentage (84.3 %).

**Table 3:** Summary of the respondents’ perceptions on the mode of transmission and preventive actions of zoonoses, and the risky practices for contracting zoonoses

Variable	Response	Cattle owners (*n* = 102)		Commodity Chain workers (*n* = 33)		Health workers (*n* = 58)		Total (*n* = 193)	
		*n*	%	*n*	%	*n*	%	*n*	%
Transmission	Contact	9	8.8	23	69.7	39	67.2	71	36.8
	Bite	11	10.8	8	24.2	26	44.8	45	23.3
	Consumption	48	47.1	28	84.8	47	80.0	123	63.7
Risk factor	Consume meat	102	100	32	97	56	96.6	190	98.4
	Consume game meat	86	84.3	17	51.5	26	44.8	129	66.8
	Consume raw meat	23	22.5	6	18.2	3	5.2	32	16.6
	Consume meat from informal sources	46	45.1	3	9.1	16	27.6	65	33.7
	Consume milk	101	99.0	30	90.9	55	94.8	186	96.4
	Consume raw milk	54	52.9	9	27.3	17	29.3	80	41.5
	Consume milk from informal sources	13	12.7	5	15.2	16	27.6	34	17.6
Preventative action	Reason for cooking meat as fear of zoonoses	36	35.3	15	45.5	33	56.9	84	43.5
	Reason for boiling milk as fear of zoonoses	33	32.4	9	27.3	29	50.0	71	39.4

Of the total respondents, 33.7 and 17.6 % indicated that they obtained meat and milk from informal sources, respectively. The proportion of respondents aware of cooking meat or boiling milk as preventative methods of zoonosis was highest for health workers compared to the other two respondent categories.

### Behavioral risk factors of getting infection in relation with awareness of zoonoses, brucellosis and tuberculosis

**Fig. 2:**
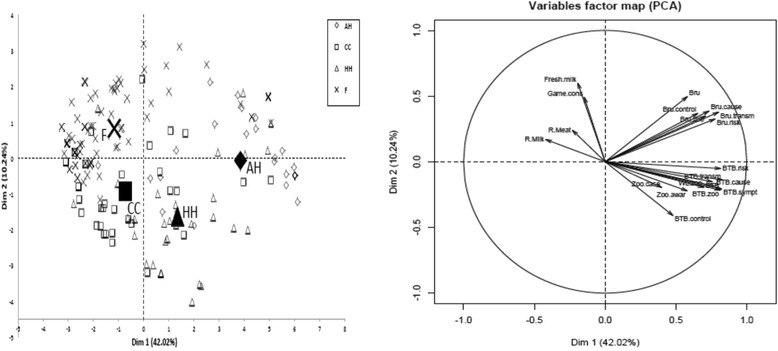
(LEFT). The individual factor map shows the different respondent groups, Animal Health workers (AH), Commodity Chain players (CC), Cattle owners (F) and Human Health workers (HH). (RIGHT) Showing the individual factors associated with famers’ awareness and perceptions and practices that put them at risk of contracting zoonoses

Figure 2 shows the results of the principal component analysis (PCA), with Fig 2a indicating the respondents factor map for the different respondent groups; animal health workers (AH), commodity chain players (CC), cattle owners (F) and human health workers (HH), while Fig 2b shows the variable factor map. Cattle owners were grouped mainly in the upper left quadrant whilst a small proportion of them were grouped in the upper right quadrant. Animal health workers were mainly grouped on the right side of the map. Human health workers were scattered on the map with a slightly higher percentage in the lower-right quadrant. Concerning the commodity chain workers they were mainly located in the lower-left quadrant. When the respondents map is superimposed with variable factor map, showed that access to fresh milk and consumption of raw milk and game meat were closely linked with cattle owners on the upper left quadrant. On the opposite side and lower right quadrant (negatively correlated) awareness of zoonoses was linked with bTB awareness and its characteristics (aetiology, zoonotic implications, mode of transmission, risk factors, preventive and control measures). Regarding the first principal component (X-axis) the risk of contracting zoonoses mainly through consumption of raw milk was also opposite to brucellosis awareness and the knowledge of its characteristics. On the Y-axis, access to fresh milk and game consumption was very close to brucellosis awareness and its characteristics. The circle of correlation of the principal component analysis, drawn with the first and second principal components, explained 52.3 % of the total inertia.

## Discussion

The apparent low proportion of cattle owners aware of zoonoses observed in this study is consistent with earlier studies on pet owners’ [9], dairy farmers’ [10] and rural cattle owners’ [11] awareness in the country. In a study by Mosalagae and co-workers [10], 55.9 % of dairy farmers from smallholder and commercial farms were aware of cattle zoonoses compared to 48 % of cattle owners in the current study at the selected wildlife-domestic animal-human interface areas.

While the differences in awareness to zoonoses among the different groups of respondents may generally be linked to the level of education and training received, it is probable that other extenuating factors may be involved since a certain proportion of health workers were not aware. The low proportion of cattle owners that were aware of zoonoses could be attributed to limited education on zoonoses since only a low number of the respondents received education on zoonoses. We noted that continuing education campaigns on awareness of zoonoses and inter-sectoral communications between veterinary and human medical workers are infrequent probably due to low numbers of trained personnel and limited resources in these rural centers. In two separate studies of community awareness on zoonoses and assessment of awareness of food-borne zoonoses in relation to providing veterinary public health services in Ethiopia, it was also demonstrated that public health centres did little in terms of providing public health services through increasing awareness to zoonoses [24, 25]. This low community awareness could be contributed by lack of access to local data on zoonoses and inadequate communications between veterinary and human medical professionals as explained by Cripps [26] in Tanzania. The cattle owners in our study are situated in agro-ecological region V which is unsuitable for crop agriculture and other commercial farming activities and these circumstances are likely to disadvantage the communities in terms of access to specialist agricultural services such as information on animal health including zoonotic infections. It is noteworthy that cattle owners from the high interface areas showed a significantly lower awareness of zoonoses as compared to their counterparts from both medium and low interface areas. Although in this area, the proportion of cattle owners who had received secondary education was lower than others, presumably because of the negative perceptions about the role of education in development by some ethnic groups in this high interface area [21], but education alone cannot explain the differences in awareness. Other than limited education alone, other factors such as lack of ready access to alternative sources of information on zoonoses from the mass media (radio, television and newspapers) that are readily accessible to cattle owners in the urban and peri-urban areas of Chiredzi town and Chikombedzi areas may explain the differences. The observed high proportions aware of zoonoses (over 80 %) amongst the health workers and commodity chain players irrespective of their areas of origin could be attributed to their level of education as well as the nature of their work that enables them to access information on zoonoses readily.

Cattle owners, commodity chain players and health workers cited anthrax, rabies, tuberculosis and brucellosis as major zoonoses. This supports earlier findings from other parts of Zimbabwe [10, 11] and other countries in the Sub-Saharan region [7, 8, 25, 27, 28] that showed anthrax, tuberculosis and rabies as the most frequently mentioned zoonoses not withstanding a lower awareness of brucellosis as a zoonosis. The observed high proportions aware of these specific zoonoses could be attributed to regular (often annual) vaccination campaigns that are conducted by the Department of Livestock and Veterinary Services countrywide. The relatively high awareness of tuberculosis and brucellosis and their zoonotic implications by the health workers could be attributed to the deliberate drive by policy makers in the respective sectors to control the two neglected tropical diseases in response to a global initiative to eradicate them [29]. In Zimbabwe, there has been an increase on the prevalence and incidence of human tuberculosis (both pulmonary and extra-pulmonary) that is associated with the HIV/AIDS pandemic. In contrast, the low awareness of tuberculosis and brucellosis in cattle owners may be explained by the fact that bovine tuberculosis has not been confirmed in cattle [14] and that the prevalence of brucellosis in cattle is low [6] in the current study areas, and is difficult to recognize clinically. However, as alluded to by de Garine-Wichatitsky and co-workers [30] increased interaction between veterinary personnel and cattle owners either through research or extension services in the study areas since the year 2007, is likely to have resulted in the improvement of the awareness of these neglected zoonoses. As was observed by Brook and McLachlan, [31] in North America, Munyeme et al., [13] in Zambia and in Tanzania [7], farmers’ awareness of disease was observed to be lower in low-prevalence settings and coupled with minimal or non-existent education campaigns compared to high-prevalence settings.

Consistent with earlier reports in Ethiopia [25], Tanzania [28] and Zimbabwe [10, 11], this study showed that consumption of animal products such as meat and milk were perceived by farmers as the primary route of transmission of zoonoses. The emphasis by health professionals to minimize food-borne zoonoses especially anthrax and brucellosis could also account for the perception that the major means of transmission of zoonoses is through consumption of contaminated animal products. Similarly, and in support of an earlier observation by de Garine-Wichatitsky et al. [32], about 46.1 % of our respondents indicated that wildlife acted as reservoirs of zoonotic infections. Considering that practices such as consumption of raw meat, raw milk and game meat are prevalent in these communities, then the potential of transmission of zoonoses cannot be discounted since brucellosis and tuberculosis have been reported in cattle and wildlife, respectively [14, 33].

Views expressed by respondents on their consumption habits for meat and milk could be inaccurate as this information was gathered mainly from males accounting for 77.2 % of the respondents. In general in Zimbabwe females are responsible for meal preparation. In a similar study involving smallholder dairy farmers in Gokwe [10], it was noted that males (especially fathers) as heads of families would want to take the prerogative of responding to the questions (from “strangers”) instead of allowing women to do so. This is in spite of the fact that women had more information about the dairies since they were running them on a daily basis. Given this scenario, further research, especially involving women who are generally responsible for meal preparation, is required.

In light of the results that do not seem logical, it has to be noted that individuals may exhibit paradoxical behaviour that is where one may be aware and not take precautionary measures [34]. In this study, three possible reasons that might be responsible for the weak relationship between risk perception and personal actions may be postulated. For instance, while some respondents in the current study may understand that wildlife play a role in transmission of zoonoses (46.1 %) yet they still consume (67 %) game meat. First, due to the close proximity to the GNP, the study areas lie in a game rich area, especially in Malipati and the majority of the population is resource-constrained. Here, the inadvertent source of the interface has been facilitated by the presence of a broken and unrepaired FMD control fence, both as animals move into the park in search of grazing [35] and partly due to human activities. Thus, game meat becomes a readily available source of animal protein. Second, the poor socio-economic standing, limited education and lastly, the lack of alternatives for the majority of cattle owners in the study areas could help explain the continued consumption of meat or milk that are obtained from the informal sources. In the high interface area of Malipati, there was no formal source for meat and respondents indicated that people from this area relied on meat obtained from their own herds, Game Park or their neighbours.

## Conclusion

The results of this study demonstrated that there was low awareness of zoonoses for most cattle owners, particularly at the high interface areas, while there was a higher awareness among commodity chain and health workers. Anthrax, rabies, tuberculosis and brucellosis were cited by all respondents as important zoonoses, except that for brucellosis, the awareness was low. We noted inadequate precautionary measures that increased the risk of contracting zoonoses among the cattle owners to be consumption of raw meat, including game meat and raw milk from informal market sources. In the high interface areas, since access to game meat and milk was closely associated with consumption of raw meat and raw milk, people living in these marginalised communities remained at high risk of exposure to zoonotic infections, given their limited education. Thus, by strengthening links and promoting inter-disciplinary “one health medicine” through carrying out awareness campaigns on zoonoses could help reduce the public health implications of zoonotic infections in human-domestic animal-wildlife interface areas.

### Ethical approval

The approval of the scientific content and merit of the proposal was made by the Scientific Steering Committee of the Research Platform-Production and Conservation in Partnership (RP-PCP), TREP Building, University of Zimbabwe, and the Faculty of Veterinary Science Higher Degrees Committee. The ethical approval to interview the people was obtained from the Department of Epidemiology and Public Health, Ministry of Health and Child Care, Harare, Zimbabwe.
